# The role of primary cerebrovascular cilia as novel sensory and signaling hubs for AD/ADRD risk factors

**DOI:** 10.1002/alz70861_108653

**Published:** 2025-12-23

**Authors:** Clare Sunderman, Wissam Aboualaiwi, Kathleen Forero, Gillian Gallagher, Neela Azizi

**Affiliations:** ^1^ University of Toledo, Toledo, OH USA

## Abstract

**Background:**

Primary cilia are microtubule‐based organelles, extending from the surface of vascular endothelial cells to sense extracellular signaling cues and fluid‐shear stress. Cilia dysfunctions (ciliopathies) manifest a broad range of symptoms, including vascular dysfunction, hypertension (HTN) and cognitive and memory dysfunction. We demonstrated that the inability of primary endothelial cilia to sense blood flow can lead to nitric oxide (NO) deficiency and cause vascular dysfunction and HTN. HTN in turn, can cause brain microvascular endothelial mechanical stress and ultimately induce cognitive impairment and Alzheimer’s disease (AD). In addition, decreased biosynthesis of NO contributes to impaired microvascular function in AD patients through increased deposition of beta amyloid (Aβ). However, the molecular mechanisms underlying the pathogenesis of impaired vascular function in AD are incompletely understood thereby limiting our ability to prevent progression of AD. We hypothesize that *primary cilia serve as novel sensory and signaling hubs for AD risk factors* and that *altered ciliary signaling contributes to cerebrovascular dysfunction and AD*.

**Method:**

We examined the pathophysiological roles of cerebrovascular ciliary receptors in vascular function, BP, and AD. This is in support of the concept that in early stages of AD, diminished cilia‐mediated NO biosynthesis and deposition of endothelium‐derived Aβ in cerebral blood vessels is an important mechanism contributing to the pathogenesis of impaired cerebrovascular function, HTN, and AD. Interestingly, mouse models we created characterized by EC‐specific deletion of M3R and IFT88 (ciliogenesis) developed altered vascular reactivity, increased BP associated with attenuated NO production, and impaired cognitive function.

**Result:**

We utilized human brain microvascular endothelial cells (HBECs) to examine how distorted cilia structure, impaired signaling, and increased Aβ levels, contribute to diminished Ca^+2^ and NO release, and demonstrated that pharmacological modulation of cilia structure and function rescued cilia‐mediated signaling. Furthermore, we examined the role of M3R KO in BP and AD manifestations in the *3xTgAD* model and demonstrated that vascular‐specific M3R KO worsen BP and cognitive dysfunction in AD.

**Conclusion:**

This project offers new opportunities to utilize endothelial M3R as target for therapeutic interventions designed to prevent detrimental effects of HTN on cognitive function.

